# Asymmetric projections of the arcuate fasciculus to the temporal cortex underlie lateralized language function in the human brain

**DOI:** 10.3389/fnana.2015.00119

**Published:** 2015-09-15

**Authors:** Shigetoshi Takaya, Gina R. Kuperberg, Hesheng Liu, Douglas N. Greve, Nikos Makris, Steven M. Stufflebeam

**Affiliations:** ^1^Athinoula A. Martinos Center for Biomedical Imaging, Massachusetts General Hospital and Harvard Medical SchoolCharlestown, MA, USA; ^2^Department of Psychology, Tufts UniversityMedford, MA, USA; ^3^Harvard-Massachusetts Institute of Technology Division of Health Sciences and TechnologyCambridge, MA, USA

**Keywords:** language pathway, cortical projection, structure-function, surface-based, structural connectivity, functional connectivity, MRI, semantic decision

## Abstract

The arcuate fasciculus (AF) in the human brain has asymmetric structural properties. However, the topographic organization of the asymmetric AF projections to the cortex and its relevance to cortical function remain unclear. Here we mapped the posterior projections of the human AF in the inferior parietal and lateral temporal cortices using surface-based structural connectivity analysis based on diffusion MRI and investigated their hemispheric differences. We then performed the cross-modal comparison with functional connectivity based on resting-state functional MRI (fMRI) and task-related cortical activation based on fMRI using a semantic classification task of single words. Structural connectivity analysis showed that the left AF connecting to Broca's area predominantly projected in the lateral temporal cortex extending from the posterior superior temporal gyrus to the mid part of the superior temporal sulcus and the middle temporal gyrus, whereas the right AF connecting to the right homolog of Broca's area predominantly projected to the inferior parietal cortex extending from the mid part of the supramarginal gyrus to the anterior part of the angular gyrus. The left-lateralized projection regions of the AF in the left temporal cortex had asymmetric functional connectivity with Broca's area, indicating structure-function concordance through the AF. During the language task, left-lateralized cortical activation was observed. Among them, the brain responses in the temporal cortex and Broca's area that were connected through the left-lateralized AF pathway were specifically correlated across subjects. These results suggest that the human left AF, which structurally and functionally connects the mid temporal cortex and Broca's area in asymmetrical fashion, coordinates the cortical activity in these remote cortices during a semantic decision task. The unique feature of the left AF is discussed in the context of the human capacity for language.

## Introduction

Asymmetric organization of structural and functional network is a fundamental characteristic of the brain, on which a variety of cognitive process depends (Geschwind and Galaburda, 1985; Toga and Thompson, 2003; Liu et al., 2009). Although the anatomical and functional difference between hemispheres are observed in many other mammals than humans, the lateralized specialization, especially in language-related network, is thought to develop during the course of evolution and development (Buxhoeveden et al., 2001; Schenker et al., 2010; Friederici et al., 2011; Perani et al., 2011). Investigating asymmetry in structural network and its functional relevance may thus provide insights into better understanding of human brain evolution and development. Previous studies have demonstrated a general correspondence between structural and functional network in the human brain (Koch et al., 2002; Hagmann et al., 2008; Skudlarski et al., 2008; Honey et al., 2009). A more recent study has shown that the structural connectivity of the right fusiform gyrus with the whole brain predicts functional activation to face in this region (Saygin et al., 2012). However, the structure-function relationship of a specific fiber pathway in the context of the specialized cortical function in the human brain remains poorly understood. Given functional operation in the brain is done with the interaction between the remote cortices through the white matter pathway, an asymmetric white matter pathway is likely to be related to the functional lateralization of the cortex.

The arcuate fasciculus (AF), or dorsal language pathway, is a major association pathway that shows a remarkable asymmetry. This pathway interconnects the anterior and posterior language-related cortices and has been considered a key component of the human language network for a century (Wernicke, 1874; Déjèrine, 1895; Geschwind, 1970). The recent advent of diffusion MRI tractography enables us to trace the trajectory of the AF in the living human brain. Studies using this technique have shown that the white matter volume of the parietal-frontal AF pathway (the anterior segment of the AF, Catani et al., 2005 or the SLF III, Makris et al., 2005) is larger in the right hemisphere, while that of the temporal-frontal AF pathway (the long segment of the AF, Catani et al., 2005) is larger in the left hemisphere (Parker et al., 2005; Powell et al., 2006; Catani et al., 2007; Thiebaut de Schotten et al., 2011). The AF terminates anteriorly into the *pars triangularis* and *pars opercularis* in the inferior frontal lobe (Broca's area in the left hemisphere), although additional projections to adjacent frontal areas have also been recognized (Catani et al., 2005; Frey et al., 2008; Glasser and Rilling, 2008; Rilling et al., 2008; Thiebaut de Schotten et al., 2012). In contrast, the posterior origin of the AF is more extensively dispersed within the inferior parietal and lateral temporal cortices and shows a remarkable asymmetry in the human brain (Powell et al., 2006; Rilling et al., 2008, 2011). However, the distribution of the asymmetric AF projections in the lateral temporal and inferior parietal cortices and its relevance to cortical function in the human brain remains unclear.

In the current study, we investigated the structure–function relationship of the asymmetric AF projections in the human brain using MRI. The AF was defined as the fiber bundle that originates from the inferior parietal and lateral temporal cortices and terminates in Broca's area (the *pars triangularis* and *pars opercularis*) through the dorsal rout. This definition is consonant with a classic model of the AF as a language-related pathway (Wernicke, 1874; Déjèrine, 1895; Geschwind, 1970). It also works to compare our results with non-human primate studies in which the posterior projection regions of the homologous pathway terminating in the homolog of Broca's area were explored using histochemichal tract-tracing techniques (Schmahmann and Pandya, 2006; Petrides and Pandya, 2009). We first developed surface-based structural connectivity analysis: the integration of probabilistic tractography analysis implementing a multi-fiber reconstruction algorithm (Behrens et al., 2007) and surface-based mapping/alignment (Dale et al., 1999; Fischl et al., 1999) and mapped the asymmetric distribution of the AF projections in the inferior parietal and lateral temporal cortices. This method tracks fibers to the gray-white matter boundary surface, yielding connection probability estimates of specific fiber bundles on the cortical surface in individual brains. This allowed us to perform surface-based (two-dimensional) cross-subject and cross-hemisphere (left-right) alignment to perform a statistical comparison between the two hemispheres. Moreover, this method also allowed us to directly compare these AF projections with the blood oxygen level-dependent (BOLD) signal change measured by fMRI within the same cortical space. Thus, we investigated whether asymmetrically connected cortices through the AF also show asymmetry in functional connectivity at rest measured by resting-state fMRI. Furthermore, we explored whether asymmetric connectivity of the AF is related to the left-lateralized cortical activity during a language task. For this purpose, we also acquired fMRI data using a semantic classification task of single written words that is known to evoke markedly asymmetric activation in Broca's area and the lateral temporal cortex and thus has been used to identify language-dominant hemisphere in presurgical evaluation (Demb et al., 1995; Wang et al., 2014).

## Materials and methods

### Subjects

Twenty-five right-handed healthy subjects participated in the current study (17 females and 8 males; age range, 18–31 years; mean age, 22.9 years). The study was approved by the institutional review board of Massachusetts General Hospital and each subject provided written informed consent.

### Imaging data acquisition

All images were acquired on a 3 Tesla Siemens TimTrio scanner (Erlangen, Germany). A high-resolution three-dimensional structural image was acquired using the magnetization-prepared rapid-acquisition gradient-echo (MPRAGE) sequence (voxel size: 1 × 1 × 1 mm; repetition time (TR): 2000 ms; echo time (TE): 3.37 ms; flip angle: 10°). Diffusion-weighted data were acquired using echo planar imaging (voxel size: 2 × 2 × 2 mm; diffusion weighting, isotropically distributed along 60 directions; b value: 700 s/mm^2^). Two runs of resting-state fMRI data were acquired using the gradient echo sequence (voxel size: 2 × 2 × 2 mm, TR: 5000 ms, TE: 30 ms, flip angle: 90°, acquisition duration: 380 s). Subjects were instructed to stay awake and look at the crosshair; no other task instruction was provided.

Three runs of task-related fMRI data were acquired using the gradient echo sequence (voxel size: 3 × 3 × 3 mm; TR: 2000 ms; TE: 30 ms; flip angle: 90°; slice gap: 0.6 mm). We used a semantic classification task of single written words (Demb et al., 1995; Liu et al., 2009; Whitney et al., 2011). Each run of task-activation fMRI data consisted of one 8-s initial block that was discarded to allow for T1-equilibration effects in data analysis, followed by a 28-s block of fixation and then a 36-s block for the task. There were three such fixation/task blocks in each run. During the task blocks, 12 written words (six concrete and six abstract words) were presented in random order for 2 s each with a 1-s interstimulus interval. In total, 108 stimuli were presented. Participants were asked to indicate if the word was concrete or abstract without articulating. They were instructed to indicate their response by pressing a key on a keyboard (left-hand key press for abstract words, right-hand key press for concrete words).

### Structural connectivity

#### Cortical regions of interest

**Figure 1:**
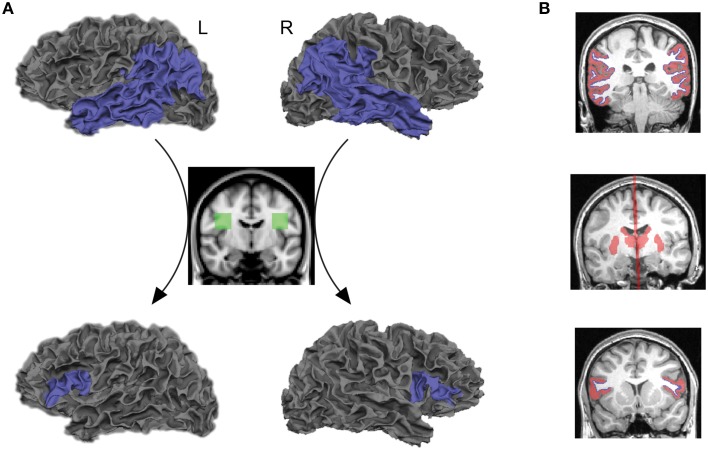
**Seed and target regions, as well as inclusion and exclusion masks for tractography and structural connectivity analyses**. **(A)** Probabilistic tractography was performed by starting the tracts from the gray–white matter boundary surface of the posterior language-related area in the left hemisphere or its right homolog (blue areas, top row) and terminating them at the boundary surface of Broca's area or its right homolog (blue areas, bottom low). Only those tracts that passed through the white matter inclusion mask in each hemisphere (green areas, middle low) were selected for tractography analysis. Also see Supplementary Figure 1. **(B)** The subcortical structures including the thalamus and striatum, and the midline sagittal plane were used as exclusion masks (red areas, middle row). The gray matter volumes attaching the gray–white matter boundary surface of seed and target regions were also used as exclusion masks (red areas, top, and bottom rows). The gray–white matter boundary surface of seed and target regions are displayed in blue.

The posterior language-related area and Broca's area in the left hemisphere, as well as the homologous regions in the right hemisphere, were extracted from each individual cortex using an automated parcellation process based on the Desikan–Killiany cortical atlas (Desikan et al., 2006) implemented in FreeSurfer (http://freesurfer.net/). The posterior language-related area and its homologous region were liberally defined as the entire inferior parietal and lateral temporal cortices (the superior/middle/inferior temporal, supramarginal, and angular gyri) so that they included the projection regions previously proposed from human and non-human primate studies (Déjèrine, 1895, 1901; Catani et al., 2005; Schmahmann and Pandya, 2006; Petrides and Pandya, 2009; Dick and Tremblay, 2012). Broca's area and its right homolog were defined as the *pars opercularis* and *pars triangularis*. The gray–white matter boundary surface of each region of interest, provided from a FreeSurfer cortical reconstruction, were transformed into a three-dimensional anatomical space and used as seeds and targets for tractography analysis (blue areas in Figure 1A, top and bottom rows).

#### Inclusion and exclusion masks

The symmetric white matter inclusion masks were used for tracing the AF (green areas in Figure 1A, middle row). Only those tracts that passed through these masks in each hemisphere were selected for tractography analysis. The mask for the left AF was located in the white matter of a standard space (MNI 152) at the level of a single coronal slice at *y* = −8, extending from *x* = −28 to −48, and *z* = 16 to 36. The inclusion mask for the right AF was created by flipping the masks in the left hemisphere. These masks included both the segments of the AF pathway that project to the inferior parietal lobule (anterior segment) and temporal lobe (long segment), identified in a normative white matter atlas created from 40 young healthy subjects (Catani and Thiebaut de Schotten, 2012). They were registered onto the native space of each subject using linear and non-linear transformation implemented in FMRIB's Software Library (FSL) (www.fmrib.ox.ac.uk/fsl). We confirmed that these masks included fibers oriented in an anterior-posterior direction on each individual color-coded diffusion map (white outlined areas in Supplementary Figure 1).

The goal of the current study was to reveal direct connections between seed and target regions; therefore, the thalamus and striatum were extracted from each individual brain by FreeSurfer and used as exclusion masks to remove the indirect pathway that connects seed and target regions via these subcortical structures (Behrens et al., 2003; Leh et al., 2007). We also used the midline sagittal plane as an exclusion mask to reject the indirect pathway entering the opposite hemisphere (red areas in Figure 1B, middle row). Furthermore, we extracted gray matter volumes in the cortical regions of interest and used them as exclusion masks to reduce false positive tracts that are initiated from the seed and terminated in the target through the gray matter (red areas in Figure 1B, top and bottom rows).

#### Structural connectivity estimation

**Figure 2:**
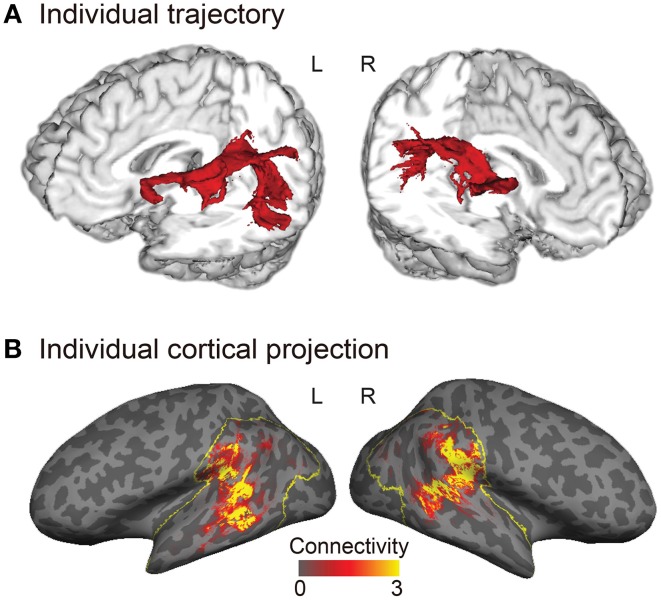
**Trajectory and cortical projections of the arcuate fasciculus (AF) in a representative subject. (A)** Trajectory of the AF reconstructed in a three-dimensional individual brain. **(B)** Normalized structural connectivity to Broca's area (or the right homolog) through the AF in the inferior parietal and lateral temporal cortices (yellow outlined regions) is displayed on the inflated surface of an individual brain. Darker and lighter regions on the inflated surface denote the sulci and gyri, respectively. The normalized structural connectivity was calculated by dividing the connection probability of each voxel in the inferior parietal and lateral temporal cortices by the total number of sample streamlines that reached Broca's area (or the right homolog), and then rescaled (× 10^4^) for display purpose.

Tractography analysis and structural connectivity estimation were performed using FMRIB's Diffusion Toolbox implemented in FSL. Diffusion-weighted images were corrected for eddy currents and head motion using affine registration to the non-diffusion volumes (*b* = 0). Probabilistic diffusion MRI tractography was performed by starting the tracts from the gray–white matter boundary surface of the posterior language-related area (or its right homolog) and terminating them at the boundary surface of Broca's area (or its right homolog). We drew 5000 samples from each voxel in each seed region and the number of tracts that reached a target region gave the connection probability. We used the standard parameters with a curvature threshold of 0.2, a step length of 0.5, and a maximum of 2000 steps. The connection probability of each voxel was then normalized by dividing it by the total number of sample streamlines that reached a target from a seed region. The normalized connection probability maps of the AF were sampled from the volume onto the inflated cortical surface of each individual's left and right hemispheres (Figure 2B).

#### Surface-based averaging and interhemispheric comparison

To generate group-averaged images of the AF projections, individual normalized connection probability maps on the cortical surface were transferred to the averaged cortical surface template of each hemisphere using surface-based alignment (Dale et al., 1999; Fischl et al., 1999). Then, smoothing was performed along the surface with a two-dimensional 10-mm full width at half maximum (FWHM) Gaussian kernel.

For interhemispheric analysis, individual normalized connection probability maps on the cortical surface of the subject's left and right hemispheres were transferred to a left–right symmetric template. This template was constructed by averaging the cortical folding pattern of both hemispheres (Greve et al., 2013). The interhemispheric comparison of the AF projections was performed using a paired *t*-test between the left and right hemispheres. Clusters were defined using a vertex-wise threshold of *p* < 0.01. Surface-based cluster-wise correction for multiple comparisons was applied using a Monte Carlo procedure (*p* < 0.01).

### Functional connectivity and cortical response during the task

#### Preprocessing for fMRI data analyses

For both resting-state and task-related fMRI data analyses, surface-based analysis was conducted using FreeSurfer Functional Analysis Stream (FS-FAST) (http://surfer.nmr.mgh.harvard.edu/fswiki/FsFast, http://surfer.nmr.mgh.harvard.edu/fswiki/FsFastFunctionalConnectivityWalkthrough). After the first four volumes were discarded to allow for T1-equilibration effects, the fMRI was motion corrected to the middle time point then slice-timing correction were performed. The middle fMRI time point was registered to the anatomical image in each subject using boundary-based registration (Greve and Fischl, 2009) and sampled onto the surface.

#### Regions of interest to extract the BOLD signal

**Figure 3:**
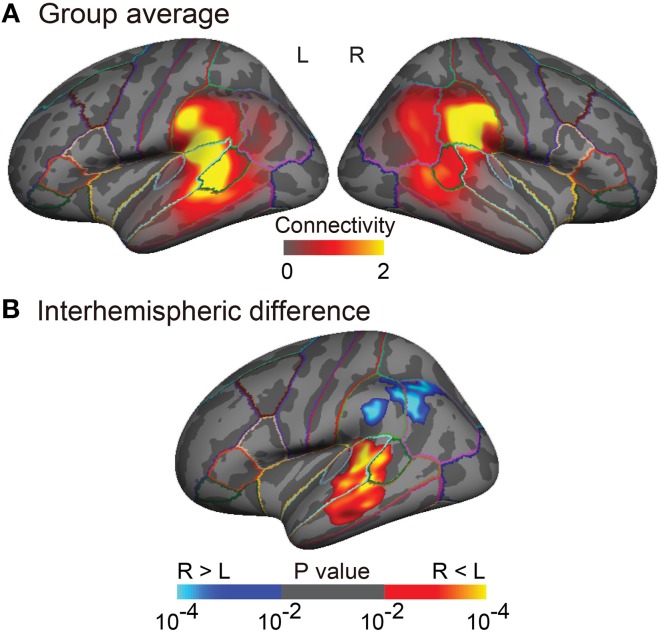
**Group analyses of the arcuate fasciculus (AF) projections in the inferior parietal and lateral temporal cortices**. **(A)** Group-averaged images (*n* = 25) of normalized structural connectivity to Broca's area (or the right homolog) through the AF in the inferior parietal and lateral temporal cortices is displayed on the inflated surface of an averaged brain. The normalized structural connectivity is rescaled (× 10^4^) for display purpose. Outlines indicate parcellations of the Desikan-Killiany atlas (Desikan et al., 2006). **(B)** Interhemispheric difference of normalized structural connectivity to Broca's area (or the right homolog) through the AF is displayed on the inflated symmetric surface (*n* = 25). A paired *t*-test was performed. Brain regions showing positive (warm colors) and negative (cold colors) values refer to the left-lateralized and the right-lateralized structural connectivity of the AF, respectively. Maps are thresholded at *p* < 0.01. Surface-based cluster-wise correction for multiple comparisons was performed at *p* < 0.01.

**Figure 4:**
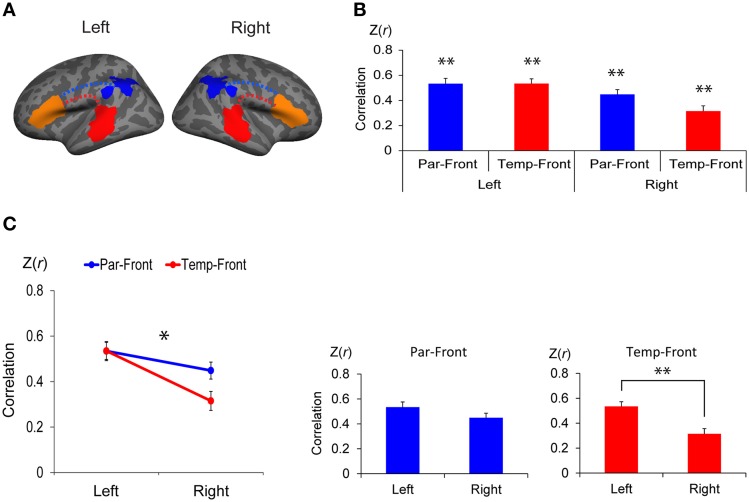
**Functional connectivity between the anterior and posterior cortical ends of the arcuate fasciculus (AF) in each hemisphere**. **(A)** Regions of interest (ROI) to extract the time course of blood oxygen level dependent (BOLD) signal for functional connectivity analysis. The clusters found in the interhemispheric comparison of structural connectivity (the left- and the right-lateralized projection regions of AF in Figure 3B) were defined in both hemispheres as the posterior ends of the AF (temporal-ROIs shown as red areas and parietal-ROIs shown as blue areas, respectively). Broca's area and the right homolog were defined as the anterior ends of the AF (frontal-ROIs shown as orange areas). **(B)** Correlations between each pair of ROIs. Correlation coefficients were transformed to z-score [Z(*r*)] using Fisher's *r*-to-z transformation. Par-Front and Temp-Front indicate functional connectivity between the parietal-ROI and the frontal-ROI, and between the temporal-ROI and frontal-ROI, respectively. Asterisks indicate the group-level significance of functional connectivity (^**^p < 0.005). Error bars indicate SEM. **(C)** A repeated measures analysis of variance (ANOVA) shows a significant interaction between hemisphere (left vs. right) and pathway (temporal-frontal vs. parietal-frontal) (line graph, ^*^p < 0.05). The *post-hoc* paired *t*-tests (bar graphs) demonstrate that the functional connectivity between the temporal-ROI and the frontal-ROI in the left hemisphere is significantly higher than that in the right hemisphere (^**^p < 0.005).

**Figure 5:**
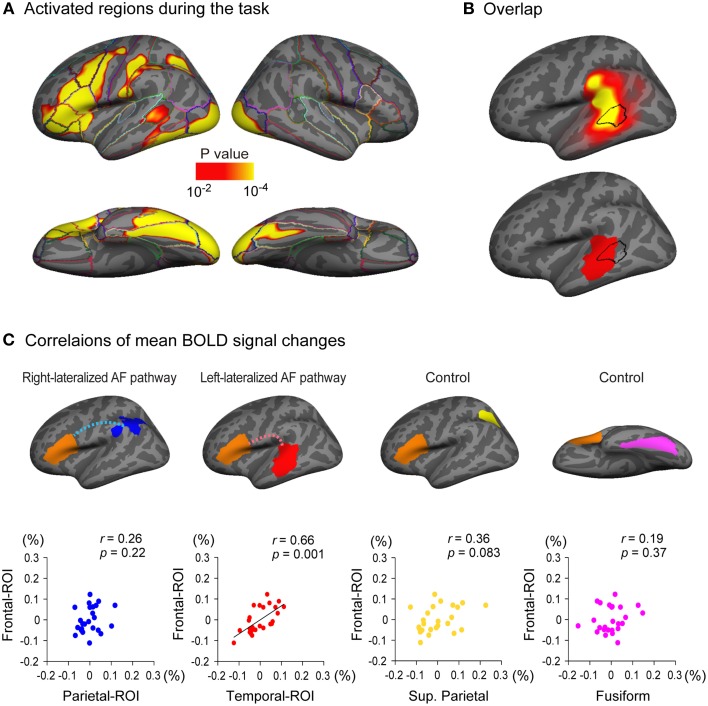
**Relationship between cortical response during a language task and the arcuate fasciculus (AF) projections. (A)** Brain regions activated during a semantic classification task of single written words relative to fixation condition (*n* = 25). A threshold of *p* < 0.01 was applied with two-dimensional cluster-wise correction for multiple comparisons (*p* < 0.01). **(B)** The activated cluster in the lateral temporal cortex during the task (black outlined area) is put on the inflated surface of an averaged brain with the cortical projection regions of the left AF (top row, also see Figure 3A) and the left-lateralized projection region of the AF (bottom row, also see Figure 3B). **(C)** Correlations of task-related responses between the posterior and anterior ends of the arcuate fasciculus (AF) are examined. The mean blood oxygen level dependent (BOLD) signal change (%) of Broca's area (frontal-ROI, orange area) during the task is plotted against that in the right-lateralized projection regions (parietal-ROI, blue area) and the left-lateralized projection regions (temporal-ROI, red area) of the left AF. Residuals after controlling for the effect of the global response are plotted. A significantly correlated response was observed between the temporal-ROI and the frontal-ROI (second scatter plot), but not between the parietal-ROI and the frontal-ROI (first scatter plot). The correlations between the mean BOLD signal change of the frontal-ROI and that of two control regions (the superior parietal lobule, yellow area; the fusiform gyrus, magenta area) were also examined to ensure that the significantly correlated response observed between the temporal-ROI and the frontal-ROI were not simply the result of activation during the task within the two ROIs. These control regions were selected because they were also activated during the task **(A)** but are not anatomically connected to Broca's area (frontal-ROI). The mean BOLD signal change of the frontal-ROI was not correlated with that of either region (third and fourth scatter plots).

The clusters found in the interhemispheric comparison of structural connectivity (the left- and the right-lateralized projection regions of the AF in Figure 3B) were defined in both hemispheres as the posterior ends of the AF (temporal-ROIs and parietal-ROIs, respectively). Broca's area and the right homolog were defined as the anterior ends of the AF (frontal-ROIs). These clusters were used to extract the BOLD signal in resting-state fMRI and task-related fMRI analyses (Figures 4A, 5C).

#### Resting-state fMRI data analysis

Resting-state fMRI data were analyzed using ROI-based correlation analysis to examine the functional connectivity between the posterior and anterior ends of the asymmetric AF. After the preprocessing, the averaged time courses of BOLD signal were extracted from the temporal-ROI (left-lateralized projection regions of the AF), parietal-ROI (right-lateralized projection regions of the AF) and frontal-ROIs (Broca's area and the right homolog) in each hemisphere of each subject (red, blue, and orange areas, respectively in Figure 4A).

A temporal band-pass filter of 0.01–0.08 Hz was applied. To remove the potential effect of motion and physiological noises that are unlikely to represent the signal of neuronal origin in resting-state fMRI data, six head motion parameters, the averaged signals from the deep white matter and cerebral spinal fluid were used as the covariates to be regressed out. Then, the residual BOLD time course was used to calculate Pearson's correlation coefficient between the temporal-ROI and the frontal-ROI and between the parietal-ROI and the frontal-ROI in each hemisphere.

The correlation coefficients were transformed to z-score [Z(*r*)] using Fisher's *r*-to-z transformation, then averaged across two runs in each subject. To assess the group-level significance of the correlation between each pair of regions, we carried out one-sample *t*-tests (Shehzad et al., 2009). We tested whether the Z(*r*) for each pair of regions is significantly different from zero. To evaluate hemispheric differences, functional connectivity [Z(*r*)] between each pair of regions was submitted to a repeated measures analysis of variance (ANOVA), with hemisphere (left vs. right) and pathway (temporal-frontal vs. parietal-frontal) as within-subject factors.

#### Task-related fMRI data analysis

##### Brain regions activated during the task

After the preprocessing, each individual image was transferred to the averaged cortical surface template of each hemisphere using surface-based alignment and smoothed along the surface with a 10-mm FWHM kernel (Dale et al., 1999; Fischl et al., 1999; Hagler et al., 2006). A general linear model was used to determine the brain regions activated in the word classification task. A boxcar function was convoluted with an SPM canonical hemodynamic response function to generate the task regressor. Six head motion parameters were used as nuisance regressors. For the group average analysis, surface-based cluster-wise correction for multiple comparisons was performed using a Monte Carlo procedure at *p* < 0.01, with Bonferroni correction for the bilateral hemispheres.

##### Correlations of task-related responses

We examined if the cortical responses evoked by the language task were correlated at both ends of the AF. The mean percent signal change during the task was extracted from the posterior and anterior projection regions of the left AF. We performed partial correlation analyses after controlling for the global response (the mean percent signal change over the entire cortex in the left hemisphere). The global response was controlled because the task was expected to induce a global effect expressed in all the regions of interest in each subject, which might create widespread, non-specific correlations between a given pair of regions of interest in cross-subject correlation analysis. In order to examine if this assumption is correct, we also performed simple correlation analyses without using a control variables.

## Results

### Asymmetric cortical projection topography of the arcuate fasciculus

The tractography and cortical projections of the AF in an individual subject are shown in Figures 2A,B. The group analysis demonstrated that the projection topography of the AF was remarkably asymmetric (Figures 3A,B). Group average maps (Figure 3A) showed that the right AF projected mainly to the cortex of the supramarginal gyrus, extending to the cortex of the angular gyrus and the most posterior part of the superior temporal gyrus (STG), superior temporal sulcus (STS), and middle temporal gyrus (MTG). In contrast, the left AF extended further inferiorly and anteriorly, projecting to the cortex of the supramarginal gyrus and the posterior STG, and extending into the cortex of the anterior part of the bank of the STS and the mid part of MTG. A statistical comparison between the two hemispheres (Figure 3B) showed that the right AF predominantly projected to a cortical region extending from the mid part of the supramarginal gyrus to the anterior part of the angular gyrus, whereas the left AF predominantly projected to a cortical region extending from the posterior STG to the mid STS/MTG.

### Functional connectivity between the posterior and anterior ends of the AF

There was significant functional connectivity in all pairs of ROIs examined (Figure 4B): between the parietal-ROI and the frontal-ROI [Z(*r*) = 0.53 ± 0.21, *t*_(24)_ = 12.49, *p* < 0.001], and the temporal-ROI and the frontal-ROI [Z(*r*) = 0.53 ± 0.19, *t*_(24)_ = 13.94, *p* < 0.001] in the left hemisphere; and between the parietal-ROI and the frontal-ROI [Z(*r*) = 0.45 ± 0.19, *t*_(24)_ = 11.76, *p* < 0.001], and the temporal-ROI and the frontal-ROI [Z(*r*) = 0.32 ± 0.21, *t*_(24)_ = 7.42, *p* < 0.001] in the right hemisphere. The results survived Bonferroni correction for multiple comparisons (*p* < 0.05/4).

A repeated measures ANOVA demonstrated a significant interaction between hemisphere (left vs. right) and pathway (parietal-frontal vs. temporal-frontal) [*p* = 0.011, *F*_(1, 24)_ = 7.70] (Figure 4C, left). The *post-hoc* paired *t*-tests revealed that the functional connectivity between the temporal-ROI and the frontal-ROI in the left hemisphere was significantly higher than that in the right hemisphere [*t*_(24)_ = 5.54, *p* < 0.001] (Figure 4C, right). The functional connectivity between the parietal-ROI and the frontal-ROI showed a marginally significant difference between hemispheres [*t*_(24)_ = 2.26, *p* = 0.033], but it did not survive Bonferroni correction (*p* < 0.05/2).

The regions of interest used in this analysis were larger than those usually used in rs-fMRI analysis (Biswal et al., 1995; Greicius et al., 2003; Van Dijk et al., 2010). In order to examine if this factor affected the results, analysis was also done using the smaller size of ROIs. The center of gravity was calculated for each significant cluster found in the interhemispheric comparison of structural connectivity. Then a 14-mm-diameter circle centered on the center of gravity was created for each cluster. Symmetric ROIs were created on the bilateral hemispheres (Supplementary Figure 2A). The averaged time course of BOLD signal was extracted from each ROI for functional connectivity analysis.

There was again significant functional connectivity between all pairs of ROIs examined (Supplementary Figure 2B): between the parietal-ROI and the frontal-ROI [Z(*r*) = 0.36 ± 0.20, *t*_(24)_ = 9.07, *p* < 0.001], and the temporal-ROI and the frontal-ROI [Z(*r*) = 0.44 ± 0.22, *t*_(24)_ = 9.82, *p* < 0.001] in the left hemisphere; and between the parietal-ROI and the frontal-ROI [Z(*r*) = 0.50 ± 0.19, *t*_(24)_ = 13.37, *p* < 0.001], and the temporal-ROI and the frontal-ROI [Z(*r*) = 0.19 ± 0.19, *t*_(24)_ = 4.91, *p* < 0.001] in the right hemisphere. The results survived Bonferroni correction for multiple comparisons (*p* < 0.05/4).

A repeated measures ANOVA demonstrated a significant interaction between hemisphere (left vs. right) and pathway (parietal-frontal vs. temporal-frontal) [*F*_(1, 24)_ = 28.96, *p* < 0.001] (Supplementary Figure 2C, left). The *post-hoc* paired *t*-tests revealed that the functional connectivity between temporal-ROI and the frontal-ROI in the left hemisphere was again significantly higher than that in the right hemisphere [*t*_(24)_ = 4.80, *p* < 0.001]. It also demonstrated that the functional connectivity between the parietal-ROI and the frontal-ROI in the right hemisphere was significantly higher than that in the left hemisphere [*t*_(24)_ = 2.73, *p* = 0.012] (Supplementary Figure 2C, right).

### Relationship between cortical response during a language task and the AF cortical projections

Left-lateralized cortical activity was observed during a semantic classification task of single written words in multiple regions including Broca's area (Figure 5A). Of particular note, the region activated in the lateral temporal cortex overlapped with the projection region of the left AF (Figure 5B, top row), especially with the left-lateralized projection region of the AF (Figure 5B, bottom row).

We further examined the correlations of the brain responses during the task in the posterior and anterior ends of the left AF. The regions from which the mean signal change was extracted in the current study were larger than those in a typical fMRI study; therefore, a preponderance of non-activated voxels could mask the activation of a small proportion of voxels in each region of interest. Thus, we excluded non-activated voxels (percent signal change of zero or less) before the mean percent signal change was calculated in each region of interest. A partial correlation analyses using the global response as a control variable revealed that the response in the temporal-ROI (left-lateralized AF projection region, red area in Figure 5C) was significantly correlated with that in the frontal-ROI (Broca's area) (partial *r* = 0.66, *p* = 0.001), which survived Bonferroni correction for multiple comparisons (*p* < 0.05/4). However, the brain response in the parietal-ROI (right-lateralized AF projection region, blue area in Figure 5C) was not correlated with that in the frontal-ROI (partial *r* = 0.26, *p* = 0.22).

To ensure that the correlated brain responses observed between the temporal-ROI and the frontal-ROI were not simply the result of activation during the task within these two ROIs, we also carried out partial correlation analyses between the response in control regions and that in the frontal-ROI. We chose the superior parietal lobule (yellow area in Figure 5C) and the fusiform gyrus (magenta area in Figure 5C) as control regions because these were also activated during the task (Figure 5A) but have not been shown to be anatomically connected to the frontal-ROI (Broca's area). The partial correlation analyses showed that the response in the superior parietal lobule and the fusiform gyrus was not correlated with that in the frontal-ROI (partial *r* = 0.36, *p* = 0.083 and partial *r* = 0.19, *p* = 0.37, respectively).

In order to examine the effect of controlling the global response, we also performed simple correlation analyses without using it as a control variable. A simple correlation analysis again showed that the response in the temporal-ROI (left-lateralized AF projection region, red area in Supplementary Figure 3) was significantly correlated to that in the frontal-ROI (Broca's area) (partial *r* = 0.62, *p* = 0.001). As we expected, when the global response was not controlled, an additional significant correlation was observed between the response in the superior parietal lobule (yellow area in Supplementary Figure 3) and that in the frontal-ROI (partial *r* = 0.53, *p* = 0.007). In addition, a marginally significant correlation was observed between the response in the parietal-ROI (blue area in Supplementary Figure 3) and that in the frontal-ROI (partial *r* = 0.44, *p* = 0.027), which did not survive Bonferroni correction (*p* < 0.05/4).

## Discussion

### Asymmetric organization of the human arcuate fasciculus projections

In the current study, we demonstrated the asymmetric distribution of the human AF projections in the inferior parietal and lateral temporal cortices using surface-based structural connectivity analysis based on diffusion MRI tractography. We used large objectively-defined (automated) seed regions that covered the entire lateral temporal and inferior parietal cortices. This enabled us to show a broad distribution of the AF cortical projections, with minimal a priori assumptions, and without being dependent on the skill and anatomical knowledge of the operator. In addition, the surface-based group analysis used here employed a cross-subject alignment based on patterns of sulci and gyri. It is advantageous to see the topographical organization of the AF cortical projections in relation to sulcal and gyral landmarks (Fischl et al., 1999; Hagler et al., 2006; Klein et al., 2010).

Previous diffusion MRI tractography studies have reported that the white matter volume of the parietal-frontal AF pathway is larger in the right hemisphere, while that of the temporal-frontal AF pathway is larger in the left hemisphere (Parker et al., 2005; Powell et al., 2006; Catani et al., 2007; Thiebaut de Schotten et al., 2011). In addition, structural connectivity of the AF in the temporal cortex is higher in the left hemisphere than the right hemisphere (Rilling et al., 2011). In the current study, we statistically compared the posterior projections of the AF between the two hemispheres and extended these previous findings by showing the distribution of the asymmetric AF projections in the inferior parietal and lateral temporal cortices in the human brain.

It is noted that the projection topography of the AF in the human right hemisphere resembled that of the homologous pathway in the macaque monkey's brain, as demonstrated using histochemical tract-tracing techniques: It originates from the homolog of the human supramarginal gyrus and angular gyrus (areas PFG and PG, respectively) and the most posterior end of the STS (Schmahmann and Pandya, 2006; Petrides and Pandya, 2009). In contrast, the projection topography of the left AF in the human brain was quite different from them in that it extended more anteriorly and inferiorly to the left mid superior temporal sulcus (STS)/middle temporal gyrus (MTG). Mapping the projections of the AF on the cortical surface enabled us to directly examine the relationship between this unique projection of the left AF and cortical function in the human brain, which may provide implications for the human capacity for language, as discussed below.

### Asymmetry in functional connectivity at rest through the AF

Functional connectivity analysis demonstrated that there was a group-level significance in functional connectivity between all pairs of the posterior ends of the AF (temporal-ROIs or parietal-ROIs) and the anterior ends of the AF (frontal-ROIs) that were examined (Figure 4B and Supplementary Figure 2B). In particular, the asymmetric extension of the left AF to the mid temporal cortex was reflected in the asymmetry of functional connectivity between these two regions (Figure 4C and Supplementary Figure 2C). A previous study has shown that each of the mid temporal cortex and the inferior frontal cortex in the left hemisphere has the stronger functional connectivity to the whole brain than that in the right hemisphere (Liu et al., 2009). Our results showed more specifically that these two regions have asymmetric functional connectivity and further demonstrated that structural asymmetry of the AF projection underlies it.

Functional connectivity between the inferior parietal cortex receiving the right-lateralized AF projections (parietal-ROI) and the anterior end of the AF (frontal-ROI) did not show asymmetry (Figure 4C). However, when we constrained the ROIs to extract the BOLD time course in the smaller regions around the center of gravity, it did show asymmetry (Supplementary Figure 2C). A study calculating the degree of functional connectivity across the brain has shown that the inferior parietal cortex works as a most prominent functional connectivity hub in the brain, namely, it has disproportionately numerous connections to the other regions (Buckner et al., 2009). In addition, functional connectivity measured by resting-state fMRI reflects both polysynaptic and monosynaptic connectivity (Lu et al., 2011), while structural connectivity in the current study mainly reflects monosynaptic connectivity. These lines of evidence suggests that the right inferior parietal cortex receiving the right-lateralized AF projections is functionally connected to the frontal cortex through the multiple routes via relaying points that are more symmetrical, whereas the central part of this region is more likely to be functionally connected to the frontal cortex through the asymmetric AF.

### Asymmetric connectivity through the AF underlies asymmetric brain activity during a language task

Mapping the AF projections on the cortex of the living human brain also allowed us to investigate the relationship between the asymmetric projection of the left AF and the left-lateralized brain activity within the cortex during a language task in the same subjects. There are several combined diffusion MRI tractography and task-activation fMRI studies that have investigated the spatial relationship between white matter pathways and the brain regions activated during language tasks (Powell et al., 2006; Glasser and Rilling, 2008; Saur et al., 2008). Among them, Glasser and Rilling have compared the projection regions of the AF extrapolated from the endpoint of the pathway in the white matter, with the activation coordinates in the cortex from different subject cohorts in prior fMRI studies (Glasser and Rilling, 2008). They reported that the projection region of the left AF in the mid part of the middle temporal gyrus overlaps the brain region activated during lexical-semantic tasks. Our results confirmed their findings by comparing the projection region of the AF and the activated region during a semantic classification task within the same cortical space of the same subject cohort.

Furthermore, we observed the unique finding that the brain responses during the task were correlated across subjects in the temporal cortex and Broca's area that were connected through the left-lateralized AF. The significance of this observation needs to be further explored. In particular, whether neural activity in these cortical regions interacts during the task in each subject remains unclear. However, considering that the correlated responses were selectively observed in the cortices that are structurally and functionally connected through the left-lateralized AF, it is reasonably assumed that this pathway was involved in the coordination of brain activation in these two regions during the semantic-decision task.

Examining patients with brain lesions has contributed to explore the involvement of a specific white matter pathway in cognitive processing (Wernicke, 1874; Geschwind, 1965). Although the lesion method continues to provide valuable information regarding structure–function relationship in the human brain network, it has some limitations to infer the function of a specific white matter pathway in the human brain (Rorden and Karnath, 2004). For example, it is rare to find a patient with a lesion limited to one specific white matter pathway; thus, the lesion may directly and indirectly affect adjacent pathways as well as the cortex. In addition, the brain can reorganize and may use alternative pathways after one pathway is injured. Mapping the projections of a specific white matter pathway on the cortex that was used in the current study may thus be advantageous in that we can directly compare them to cortical function in the healthy brain.

### Implications for human language development and evolution

The unique feature of the left AF pathway extending to the mid temporal cortex in the human brain is discussed in the context of language development and evolution. Cross-sectional and longitudinal studies of the human brain have shown that the integrity of the left temporal-frontal AF pathway increases during language development (Giorgio et al., 2010; Brauer et al., 2011; Perani et al., 2011; Yeatman et al., 2012). In addition, comparative studies using diffusion MRI tractography have shown that unlike humans, chimpanzees and macaques do not have the white matter pathway that connects the mid temporal cortex and the frontal lobe through the dorsal route (Rilling et al., 2008, 2011). The human left mid STS/MTG, in particular, is thought to play a critical role in lexical-semantic processing, acting to map conceptual representations onto syntactic representations and individual word forms (Rissman et al., 2003; Hickok and Poeppel, 2007; Lau et al., 2008). Given that such mappings are critical for all aspects of language comprehension and production (Jackendoff, 2003; Baggio and Hagoort, 2011), Rilling et al. have hypothesized that the extension of this pathway into the mid temporal lobe in the human brain is relevant to the evolution of language (Rilling et al., 2008, 2011). Our results demonstrated that the human AF projecting to the left mid temporal cortex has asymmetric structural and functional connection to the frontal cortex (Broca's area) and is involved in the left-lateralized brain activation during language-related processing that requires semantic decision. Therefore, our results extend their hypothesis and raise the possibility that the left AF in the human brain that structurally and functionally connects the mid temporal and the frontal cortices in asymmetrical fashion may underlie the human capacity for language.

### Caveats and future studies

There are some caveats to our findings. For example, tractography starting from the gray-white matter boundary surface might be subject to influence from the folding pattern of the cortex. The orientations of fiber bundles running beneath sulcal fundi are approximately parallel to the surface, and some of the fibers diverge to the cortex in a sharper angle than fibers running beneath gyral crowns (Van Essen et al., 2013). This can lead to the underreporting of tractography results in sulcal regions. On the contrary, the reconstructed tracts passing just beneath the sulcus might be trapped at the voxel edges protruding into the white matter before they reach the actual white matter surface in the gyrus. This can result in the overreporting of tractography results in sulcal regions. However, the topography of the cortical projections in our study did not show a remarkable tendency of the AF projections to preferentially land on gyral regions or sulcal regions. The effects of these bias might be minor or canceled out each other.

In addition, our sample size did not allow us to assess the effect of sex. Previous studies have reported that, compared with males, females have less hemispheric difference in the white matter volume of the temporal-frontal AF pathway (Catani et al., 2007) and show more symmetric cortical activation during language processing (Shaywitz et al., 1995). Therefore, the female-male ratio in the current study (17 females and 8 males) was likely to provide conservative results on the asymmetry of the AF cortical projection topography and functional activation during the language task. It should be noted, however, that the current study was not designed to investigate sex differences. Future studies should address the effects of sex, age, and handedness on the structural organization of AF projections in the cortex.

In the current study, we used a simple paradigm in the fMRI study to identify functional activation, with a semantic decision task and low-level non-linguistic fixation baseline. This resulted in robust brain activation in many cortical regions that mediate multiple levels of language processing. Further studies are needed to investigate the relationship between AF cortical projections and specific aspects of language processing.

Despite these limitations, we feel that the surface-based structural connectivity analysis used here may provide potential for future studies to examine changes in functional interactions between two remote cortices that are connected through specific white matter pathways, during the course of development and multiple neuropsychiatric disorders. Studies are now warranted for patients with temporal lobe epilepsy and schizophrenia, which are both characterized by structural and functional abnormalities within and across the frontal and temporal cortices (Voets et al., 2006; Kuperberg et al., 2007; Powell et al., 2007; Takaya et al., 2009; Catani et al., 2011).

## Conclusions

Using surface-based structural connectivity analysis based on diffusion MRI tractography, we demonstrated the asymmetric distributions of the AF projections in the lateral temporal and inferior parietal cortices. The mid temporal cortex and Broca's area are connected through the left-lateralized AF. These cortical regions also have a leftward asymmetry in functional connectivity at rest, and are co-activated during a semantic decision task. The unique feature of the left AF that structurally and functionally connects the mid temporal cortex and Broca's area in asymmetric fashion may be relevant to the human capacity for language.

### Conflict of interest statement

The authors declare that the research was conducted in the absence of any commercial or financial relationships that could be construed as a potential conflict of interest.
