# Supplementary material for: Asymmetric projections of the arcuate fasciculus to the temporal cortex underlie lateralized language function in the human brain
Source: Front Neuroanat. 2015 Sep 15;9:119. doi: 10.3389/fnana.2015.00119 (PMC4569731; doi:10.3389/fnana.2015.00119)
Supplement: Supplementary file 1 [file Image1.PDF]

Supplementary Figures

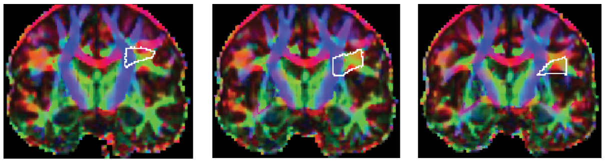

Supplementary Figure 1. White matter inclusion masks.

White matter inclusion masks (green areas in **Figure 1A**) created in a standard space (MNI 152) were transformed to the native space of each subject using linear and nonlinear transformation and registered onto color-coded diffusion maps (white outlined areas). The AF pathways running in an anterior–posterior direction (green) are included in the masks.

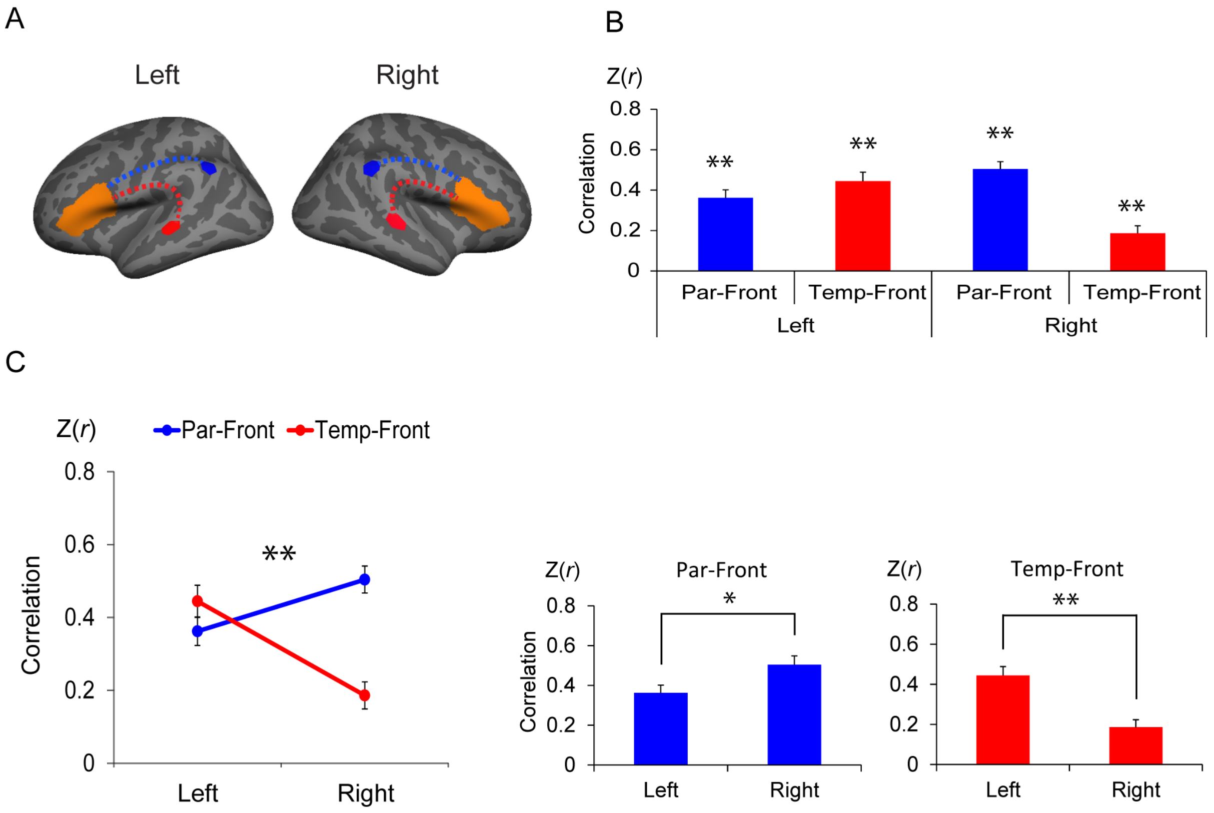

Supplementary Figure 2. Functional connectivity analyses of both cortical ends of the arcuate fasciculus (AF) using the center of gravity.

(A) Broca's area and the right homologue (frontal-ROIs) are displayed in orange. The center of gravity was calculated for each significant cluster found in the interhemispheric comparison of structural connectivity (**Figure 3B**) and expanded to a 14-mm-diameter circle. The left-lateralized and the right-lateralized projection regions of the AF are displayed in red areas (temporal-ROIs) and blue areas (parietal-ROIs), respectively. These ROIs were used to extract the time course of blood oxygen level dependent (BOLD) signal for functional connectivity analysis.

(B) Correlation between each pair of ROIs. Correlation coefficients were transformed to z-score (Z (r)) using Fisher's r-to-z transformation. Asterisks indicate the group-level significance of functional connectivity (**$p < 0.005$). Error bars indicate SEM.

(C) A repeated measures analysis of variance (ANOVA) shows a significant interaction between hemisphere (left vs. right) and pathway (temporal−frontal vs. parietal−frontal) (line graph, **$p < 0.005$). The post hoc paired *t* tests (bar graphs) demonstrate that the functional connectivity between the temporal-ROI and the frontal-ROI in the left hemisphere is significantly higher than that in the right hemisphere (**$p < 0.005$). In contrast, the functional connectivity between the parietal-ROI and the frontal-ROI in the right hemisphere is significantly higher than that in the left hemisphere (*$p < 0.05$). Also see **Figure 4**.

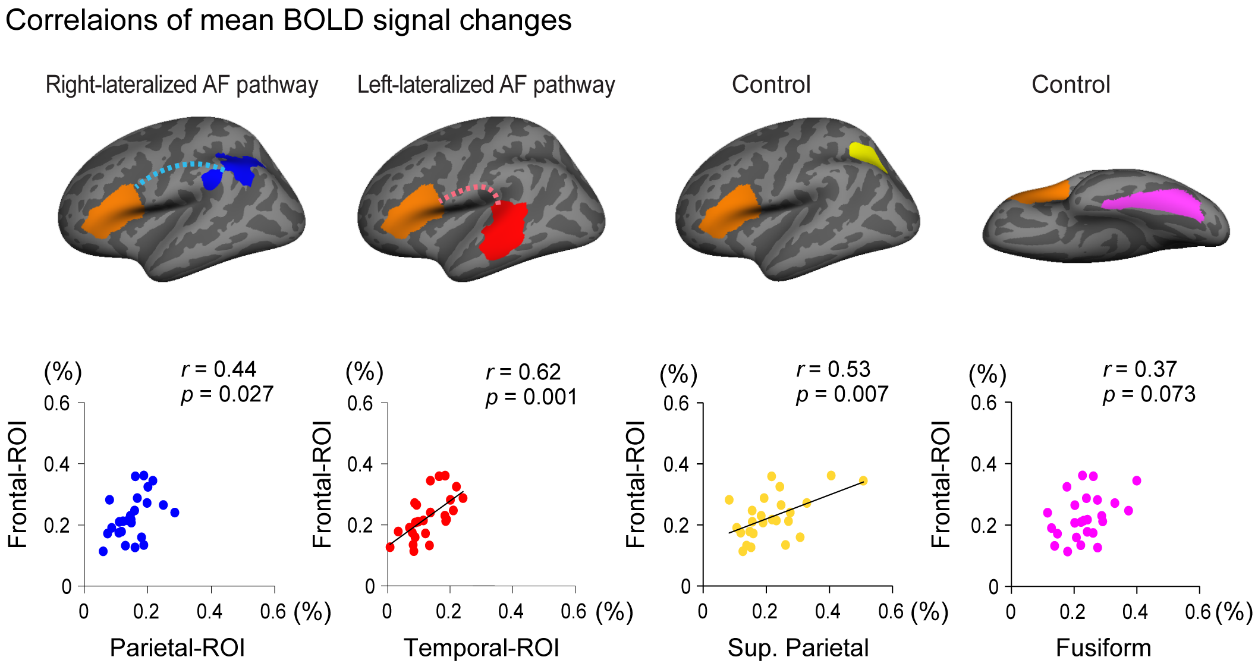

Supplementary Figure 3. Correlations of task-related responses without controlling for the global response.

The mean blood oxygen level dependent (BOLD) signal change (%) of each ROI used in **Figure 5** is plotted without controlling for the effect of the global response. An additional significant correlation was observed between the response in the superior parietal lobule (yellow area) and that in the frontal-ROI. In addition, a marginally significant correlation was also observed between the response in the parietal-ROI (blue area) and that in the frontal-ROI, which did not survive Bonferroni correction.
